# Physiological and Behavioral Factors in Musicians’ Performance Tempo

**DOI:** 10.3389/fnhum.2020.00311

**Published:** 2020-08-25

**Authors:** Shannon E. Wright, Caroline Palmer

**Affiliations:** Department of Psychology, McGill University, Montreal, QC, Canada

**Keywords:** circadian rhythms, music performance, cardiac dynamics, alertness, recurrence quantification analysis, chronotype

## Abstract

Musicians display individual differences in their spontaneous performance rates (tempo) for simple melodies, but the factors responsible are unknown. Previous research suggests that musical tempo modulates listeners’ cardiovascular activity. We report an investigation of musicians’ melody performances measured over a 12-h day and subsequent changes in the musicians’ physiological activity. Skilled pianists completed four testing sessions in a single day as cardiac activity was recorded during an initial 5 min of baseline rest and during performances of familiar and unfamiliar melodies. Results indicated slower tempi for familiar and unfamiliar melodies at early testing times. Performance rates at 09 h were predicted by differences in participants’ alertness and musical training; these differences were not explained by sleep patterns, chronotype, or cardiac activity. Individual differences in pianists’ performance tempo were consistent across testing sessions: participants with a faster tempo at 09 h maintained a faster tempo at later testing sessions. Cardiac measures at early testing times indicated increased heart rates and more predictable cardiac dynamics during music performance than baseline rest, and during performances of unfamiliar melodies than familiar melodies. These findings provide the first evidence of cardiac dynamics that are unique to music performance contexts.

## Introduction

The ways in which musical behaviors interact with human cognition and action have been of great interest to psychologists. For example, models of musical rhythm perception have posited networks of electrophysiological activity, based on populations of neuronal oscillators that fire in synchrony with musical rhythms (Large et al., 2015); these proposals suggest a tight link between musical behaviors and physiological activity. Several studies have focused on the effects of music perception on physiological measures such as heart rate and heart rate variability (see Koelsch and Jäncke, 2015 for a review). Less is known about influences of music performance on physiological processes that underlie cognition and action. We report an investigation of musicians’ melody performances measured over a 12-h day and subsequent changes in the performers’ physiological activity.

### Circadian Effects on Cognitive and Motor Performance

Several studies have documented time-of-day (circadian) effects on motor and cognitive performance. Circadian rhythms refer to approximately 24 h biological oscillations entrained to the light-dark cycle. For example, body temperature is known to fluctuate predictably over a 24 h cycle (Czeisler and Klerman, 1999), serving as a robust marker of circadian phase, and peaks and troughs in alertness tend to follow the body temperature curve (Dijk et al., 1992). Heart rate and heart rate variability (HRV) also fluctuate predictably over a 24 h period: Heart rate tends to rise in the early morning and decrease in the evening, whereas HRV is typically highest at night and lowest during the day (Bonnemeier et al., 2003; Vandewalle et al., 2007). Edwards et al. (2007) reported that participants’ improved accuracy on a simple task of flicking a counter into a target coincided with their late-afternoon peak in body temperature and alertness. A similar finding was reported by Reilly et al. (2007) for soccer-specific motor skills. Rhythmic motor tasks such as pedaling a bike (Moussay et al., 2002; Atkinson et al., 2005) and tapping a steady rhythm with one’s finger (Dosseville et al., 2002) have also been shown to have time of day effects, with peak rates of movement occurring in the late afternoon. Furthermore, Dosseville et al. (2002) found that un-cued rhythmic finger tapping rates increase as heart rate increases. Overall, these studies suggest that rhythmic motor performance is influenced by time of day effects and cardiac activity, which shows circadian rhythmicity. We address whether music performance is influenced by circadian fluctuations in physiology similar to other sequential motor activities.

Motor performance is also influenced by circadian-linked individual differences in chronotype, sleep habits, and alertness (Waterhouse et al., 2007; Tamm et al., 2009; Vitale et al., 2015). Chronotype, which depends on the phase of entrainment of one’s circadian rhythms to the light-dark cycle (Roenneberg et al., 2003a), refers to the timing of one’s sleep and wake in a 24 h period. The commonly known phenomenon of being an “early bird” or a “night owl” (Roenneberg et al., 2003b) refers to differences in the timing of the peaks and troughs of one’s circadian rhythms (Baehr et al., 2000): Early birds wake up and go to sleep earlier than night owls, and early birds are more alert in the morning and night owls more alert in the evening. Van Vugt et al. (2013) found that night owl pianists performed scales with greater temporal stability in the evening relative to the morning, while early bird pianists performed scales with more stability in the morning relative to the evening. Differences in chronotype, sleep habits, and alertness may influence performing musicians, who often work in the evening (Gjermunds et al., 2019).

### Individual Variability in Music Performance

Large individual differences in music performances of the same musical works have been documented (Palmer, 1996; Repp, 2005). One common difference across performing musicians is the tempo at which they perform a given piece. Tempo is a factor that differentiates individuals as they speak, walk, tap, and perform other rhythmic movements. These natural movement rates reflect the rate at which individuals comfortably execute a performance in the absence of external stimulus cues. Individual differences in natural movement rates have been observed not only in music performance (Loehr and Palmer, 2011; Zamm et al., 2015, 2016; Scheurich et al., 2018; Palmer et al., 2019) but also in a wide range of rhythmic movements such as walking (Hoyt and Taylor, 1981; Nessler and Gilliland, 2009), speaking (Jungers et al., 2003; Ding et al., 2017), biking (Moussay et al., 2002), and finger tapping (Fraisse, 1982; Dosseville et al., 2002). Individual differences in musicians’ spontaneous rates for simple melodies tend to be consistent within individuals but differ widely across individuals (Loehr and Palmer, 2011; Zamm et al., 2015; Scheurich et al., 2018; Palmer et al., 2019). Performers tend to drift toward their spontaneous rate in solo performances when they are initially cued at different rates (Zamm et al., 2018). Moreover, these individual differences in spontaneous rates play an important role in coordinating performances with others: pianists with similar spontaneous rates showed more synchronous performance in duets than pianists with dissimilar rates, in a variety of novel musical works (Zamm et al., 2016). Mechanisms that account for individual differences in musicians’ performance rates for the same musical works remain largely unknown; we test whether circadian-related variations in physiology can explain some of these individual differences.

### Cardiac Activity During Music Behaviors

Both the rhythms of cardiac activity and of musical behaviors form long time series of interrelated events; a few studies have addressed how heart rate modulations and musical tempo change together over time. For example, passive listening to music has shown decreased heart rate in response to slower-tempo music (Van Dyck et al., 2017) and increased heart rate during fast-tempo music (Gomez and Danuser, 2007). Heart rate variability during music listening changes less predictably; da Silva et al. (2014) found no difference in HRV between rest (baseline) and music listening, whereas Bretherton et al. (2019) reported that only some tempo manipulations elicited HRV changes relative to a rest condition. Fewer studies have examined changes in musicians’ cardiac activity as they perform. De Manzano et al. (2010) found increased heart rate as pianists played familiar music for which they reported large amounts of “flow.” Studies of performance anxiety have shown that musicians’ heart rate increased when they performed in front of an audience as compared to alone (Brotons, 1994; LeBlanc et al., 1997; Vellers et al., 2015). These studies did not, however, compare resting baseline conditions to music performance. Moreover, the impact of music performance on cardiac activity may be affected by time of day, as cardiac activity shows a circadian rhythm (Bonnemeier et al., 2003; Vandewalle et al., 2007). We investigate how cardiac activity is modulated by music performance, within and across times of day.

Despite the unfolding nature of time series for both cardiac activity and musical behaviors, most studies of heart rate and musical tempo tend to rely on linear measures that fail to capture the non-linear dynamics of the cardiovascular system and of human musical behaviors. The time series formed by music performances and cardiac activity are plausibly more complex than can be captured with a single mean value for beat-to-beat intervals or a standard deviation of those intervals. Recent studies have used non-linear methods of recurrence quantification analysis (RQA) to capture aberrant cardiac activity over time in cardiovascular patient populations (for examples, see Javorka et al., 2008, 2009; Arcentales et al., 2011). Other studies of cardiac dynamics in healthy control populations during sit-to-stand transition tasks show greater cardiac predictability during the more physically demanding standing task (Schlenker et al., 2016). Konvalinka et al. (2011) used RQA techniques to measure cardiac dynamics during a 30-min firewalking ritual during which music was heard. The cardiac dynamics became more predictable (recurrent) during the ritual than during a 30-min pre-ritual baseline measure. Goshvarpour and Goshvarpour (2012) similarly found greater predictability in cardiac dynamics during meditation than during a resting baseline state. Based on these findings, we expect that the predictability of cardiac dynamics may increase during music performance, relative to rest.

The current study had three aims. First, we investigated time-of-day effects on music performance rates by measuring musicians’ performances of simple melodies across a 12 h day while measuring their cardiac activity. To disentangle musical familiarity effects from time-of-day effects, performances of both familiar (previously learned) as well as unfamiliar (novel) melodies were measured. Second, we examined influences of circadian rhythms on individual differences in performance tempo. Based on previous findings, we hypothesized that performers with slower spontaneous rates may show slower heart rates and lower alertness than individuals with faster spontaneous rates (within the same time of day). Based on Van Vugt et al.’s (2013) study, early chronotypes were predicted to show less variable performance rates in the morning, whereas late chronotypes should show less variable performance in the evening, respectively.

Third, we investigated how the time series formed by music performance and the accompanying cardiac dynamics changed, by comparing cardiac activity during music performance with cardiac activity during a rest period. We predicted that linear measures of heart rate would be faster and HRV would be lower during music performance relative to rest. Non-linear measures of performers’ cardiac dynamics were expected to show more predictability during music performance than during rest. We also examined whether performances of unfamiliar music generated more predictable dynamics than performances of familiar music, based on previous findings of increased cardiac patterning during more demanding tasks (Javorka et al., 2008; Konvalinka et al., 2011) and increased temporal patterning in novices’ (non-musician) productions of musical rhythms than in musicians’ productions (Scheurich et al., 2018).

## Materials and Methods

### Participants

Thirty-two trained pianists with at least 6 years of private piano instruction from the Montreal community participated in the study (mean years of private instruction = 10.6; range = 6–16). Sample size was based on studies of musicians’ spontaneous performance tempo that reported moderate effect sizes for comparable samples (Palmer et al., 2019, *n* = 32 musicians; Zamm et al., 2016, *n* = 40 musicians). Participants’ mean age was 19.5 years (range = 18–27, male = 7). Twenty eight participants were right handed. Exclusion criteria included diagnosed hearing problems or sleep disorders, doing overnight shift work, habitually drinking more than three cups of coffee per day, or having taken a transcontinental flight within the 3 week period prior to participating in the study. Additionally, participants had normal hearing for the range of frequencies used in the music stimuli (<30 dB HL threshold for 125–750 Hz frequencies), as determined by audiometry screening, and had to memorize and perform short melodies without errors. Six additional participants were excluded from the study due to an inability to perform the melodies correctly from memory (3), equipment issues in collecting cardiac data (2), and having fewer than 6 years of private piano instruction (1). Participants received a small honorarium for their participation, and the study was reviewed by the Institutional Review Board of McGill University.

### Stimulus Materials and Equipment

Two musical melodies, primarily isochronous, were included in the study: Frère Jacques (“Twinkle, Twinkle,” C Major) and a Canon by Thomas Tallis (D Major). The Frère Jacques theme, composed in the 18th century, was chosen for its familiarity, whereas the Tallis canon, composed in the 16th century, was chosen for its unfamiliarity. Both musical pieces contained eight measures composed in binary (4/4) meter with the majority of quarter-note beat durations. Frère Jacques contains a few eighth notes and half notes in addition. Pianists performed each melody with their right hand, and they were provided with suggested fingerings.

Participants performed melodies on a Roland RD-700 keyboard. Participants’ auditory feedback from the keyboard was received directly through AKG K271 Studio headphones. Tones were sounded with a classical piano timbre, and the volume was set by participants to a comfortable listening level. MIDI keystroke information from the performances was recorded with FTAP (Finney, 2001) on a Dell T3600 PC running Linux (Fedora 16).

Cardiac activity was recorded with a Polar H10 heart rate monitor connected via Bluetooth to the application Elite HRV (Personal Pro) run on an iPad Mini. Sublingual temperature was measured with a digital oral thermometer (Personelle Digital Thermometer), following suggestions that sublingual temperature is a reasonable and pragmatic proxy to core body temperature under specific guidelines (Taylor et al., 2014). The temperature measures followed guidelines of a minimum measuring period of 5 min as well as ensuring the mouth is closed for the whole duration of the measurement (Pušnik and Miklavec, 2009; Taylor et al., 2014).

Alertness measures included the Psychomotor Vigilance Task (PVT) and a Visual Analog Scale (VAS). The PVT is a computer-based reaction time task in which participants are asked to click the mouse button as soon as a visual stimulus appears on the computer screen (Dinges and Powell, 1985). The 3-min version of the PVT was used, which has been previously validated (Basner et al., 2011), and presents visual stimuli at randomly varying interstimulus intervals ranging from 1 to 4 s. The PVT measures were collected on a Dell T5810 computer with a HyperX Pulsefire gaming mouse (1000 Hz polling rate) that recorded reaction times. The VAS task (Folstein and Luria, 1973; Monk, 1987) consisted of participants indicating their current level of alertness by making a vertical tick mark on a 10 cm line.

Participants completed a series of questionnaires about their sleep habits, including the Epworth Sleepiness Scale (ESS; Johns, 1991), the Pittsburgh Sleep Quality Index (PSQI; Buysse et al., 1989), and a sleep diary from Carney et al. (2012). Chronotype was measured with the Munich Chronotype Questionnaire (MCTQ; Roenneberg et al., 2003b). All participants completed the Edinburgh Handedness Inventory and a musical background questionnaire. Participants also completed a short questionnaire about their activities in the hour preceding each laboratory session that might affect alertness, body temperature, or cardiac measures.

### Design

Participants came to the lab for four testing sessions (09, 13, 17, and 21 h) in a single day. The order of testing sessions remained constant across participants (each pianist’s first session began at 9 h). Baseline physiological recordings and melody performance tasks were completed at each testing session by all participants, making this a within-subjects 4 (Testing Time) by 2 (Task: 5-min Rest/Music performance) repeated-measures design. The task order was always rest first, followed by music performance. Within the music performance task, the ordering of the familiar and unfamiliar melody performances was alternated between participants and testing sessions: Half of the participants performed the Familiar melody first at the 09 h testing session, and the other half began with the Unfamiliar melody. At subsequent testing sessions, participants alternated which melody they performed first. Each participant performed a total of 32 melody performance trials (4 times of day × 2 melodies × 3 trials) over the course of the experiment.

The main behavioral dependent variables from the melody performances were spontaneous production rate (SPR, mean interonset interval, IOI in ms) and variability of interonset intervals (measured by the coefficient of variation, SD/mean IOI). Primary physiological dependent variables included sublingual temperature (°C), heart rate (mean inter- heartbeat interval, RR), heart rate variability (measured by the standard deviation of normal-to-normal intervals, SDNN), alertness (PVT reaction times and VAS subjective scores), chronotype, and sleep deprivation measures computed from the sleep diary (described below).

### Procedure

Participants were first screened for eligibility via e-mail; if eligible, electronic copies of the musical notation (without melody titles) for the melodies used in the study were sent to participants, and participants were asked to memorize the melodies before their participation in the study. Participants also received a sleep diary which they were asked to complete for the week preceding the laboratory session.

Upon arrival at the lab, participants read and signed a consent form before completing an audiometry screening in which pure tones were presented over closed headphones (Maico MA40), to ensure they could hear the range of frequencies involved in the music performance task at a threshold of <30 db. Participants who passed the audiometry screening were invited to continue to a melody memorization task. First, participants were presented with a melody in notation. After practicing the first melody (Familiar or Unfamiliar) both with and without musical notation, participants were given up to three practice trials to perform the melody from memory without pitch errors. Then the participants repeated the task with the second melody. All participants performed the melodies without pitch errors in the memorization phase.

Next, participants attached the heart rate monitor around their chest. A 5-min baseline sublingual temperature and heart rate recording was taken during the Rest task while participants were seated and completing questionnaires. To ensure correct temperature readings, participants were instructed to insert the thermometer under their tongue and breathe normally through their nose; they were instructed to keep movement to a minimum and to avoid crossing their legs so as not to influence heart rate measures. During this time, participants marked their current alertness level in the VAS task. At the end of the 5-min rest period, participants removed the thermometer but kept the heart rate monitor on for the rest of the testing session.

Participants then completed the Psychomotor Vigilance Task. They were instructed that red numbers would appear on a black screen, and they were to click the mouse as soon as, but not before, they saw the red numbers appear. If participants clicked the mouse before the red numbers appeared, the letters “fs” appeared on the screen to inform the participant they had made a false start. A new trial was then begun. Each trial continued until participants clicked the mouse.

Participants then sat at the piano keyboard and were presented with the first melody in music notation. They were instructed to perform a practice trial consisting of four repetitions at a steady, comfortable rate without pauses. The experimenter removed the music notation and participants repeated a practice trial of the same length from memory. Once participants were comfortable with the task, they moved on to the experimental trials. Each experimental trial consisted of 4 repetitions of the melody performed from memory (in the absence of music notation) without pauses at a comfortable, steady rate. After completing all trials of the first melody, participants filled out a brief questionnaire about their activities prior to the testing session; then the same practice and experimental trials were repeated for the second melody. At the end of the melody performance task, participants removed the heart rate monitor and received a small honorarium. The same procedure was repeated at each testing session with the addition of a debriefing period at the end of the final session. The duration of the first testing session (which included the audiometric screening and memorization practice) was approximately 45 min; subsequent testing sessions took approximately 25 min.

### Data Analysis

Pitch errors in melody performances were identified by comparing the recorded MIDI data with the contents of the musical score, using the MIDI Matcher Toolbox in Matlab (Large, 1993). Repetitions containing a pitch error were excluded from analysis as timing errors are likely to co-occur with pitch errors (Drake and Palmer, 2000); 0.03% of all repetitions were excluded from analysis. The half-note durations in Frère Jacques were interpolated at the quarter-note level, and eighth notes that did not align with the quarter-note beat were excluded from the analyses. Interonset intervals (IOI), coinciding with quarter-note beats in both melodies, were computed. IOI’s greater or less than 3 standard deviations away from the mean IOI for that trial were excluded from behavioral analyses (0.13% of all IOIs).

Each participant’s Spontaneous Production Rate (SPR) was computed on the IOIs from the middle two of four melody repetitions in each trial, similar to previous studies (Zamm et al., 2016; Palmer et al., 2019), as the middle of each trial tends to show more stable tempo due to musicians’ tendencies to slow down at phrase boundaries at beginnings and endings of trials (Palmer, 1989; Repp, 1990). Participants’ SPR for each melody was then calculated from the mean IOI of the middle 2 repetitions of each trial and averaged across trials within melody. Similarly, the mean Coefficients of Variation (CV) were calculated from the same IOIs in the middle two repetitions and a mean CV was computed across trials.

Linear analyses of cardiac data were completed using Kubios (HRV Standard, 3.1.0). Mean RR intervals and the SDNN were computed for each 5-min baseline recording as well as during the total duration of melody performances, including practice and experimental trials, in order to have the longest consecutive measurement period possible. Recurrence quantification analysis (RQA) was also conducted on cardiac data using the CRP Toolbox 5.22 [Marwan, 2019, run with MATLAB 2018a (v9.4.0)]. RQA is a non-linear analysis technique, often used on behavioral and cardiac data (Javorka et al., 2008; Demos et al., 2011; Marwan et al., 2013), that identifies recurrent states in a dynamical system using Takens’s (1981) method of higher-dimensional reconstruction (Webber and Zbilut, 2005; Nayak et al., 2018). Time-delayed copies of the cardiac signals are generated and projected into multidimensional phase space (Konvalinka et al., 2011) with the parameter tau denoting the time delay. For each resting period (baseline) and music performance, tau was chosen based on the first local minimum of the average mutual information function. Tau therefore varied across participants and within participants by testing session and task (Javorka et al., 2009), and the resulting range was 2–12. The False Nearest Neighbor (FNN) method was used to select an embedding dimension; FNN values close to zero indicate that the signal is projected into a sufficient number of dimensions (Webber and Zbilut, 2005; Nayak et al., 2018). Embedding dimensions were chosen on an individual basis and ranged from 4 to 8. A Theiler window fixed to the time delay (Javorka et al., 2009) was applied to the data, as cardiac signals tend to show high autocorrelation (Martin-Gonzalez et al., 2018). Recurrence rate, the percentage of recurrent points in the system, was fixed to 5% as per previous RQA studies of cardiac signals (Javorka et al., 2008, 2009).

Recurrence plots, 2-dimensional representations of the recurrent points in a system, were generated to visualize the cardiac dynamics. Each point in the plot represents a system state that is recurrent with a previous state (Webber and Zbilut, 2005). The time series signal is plotted against itself such that the recurrence plot is symmetric across the diagonal. Two parameters were used to quantify the observed recurrence. First, determinism (DET) measured the percentage of points in the recurrence plot forming diagonal lines (excluding the line of identity), where the minimum number of points required to be considered a line was set to 2 (Equation 1). Determinism is a measure of the predictability of a system over time (Webber and Zbilut, 1994). Second, laminarity (LAM) captures the percentage of points forming vertical (or horizontal) lines in the recurrence plot (Equation 2) and is an indicator of the extent to which a system “gets stuck” in a specific state (Nayak et al., 2018).

(1) $$\%\,D\,E\,T=100*\frac{\sum_{l=l\,m\,i\,n}^{N}l\,P\,\left(l\right)}{\sum_{l=1}^{N}l\,P\,\left(l\right)}$$

(2) $$\%\,L\,A\,M=100*\frac{\sum_{v=v\,m\,i\,n}^{N}v\,P\,\left(v\right)}{\sum_{v=1}^{N}v\,P\,\left(v\right)}$$

Chronotype was determined from the MCTQ which estimates an individual’s mid-sleep point based on self-reported times of sleep onset and wake for both work and free days (Roenneberg et al., 2003b). As imposed social schedules may mask an individual’s natural mid-sleep point and lead to sleep debt (Wittmann et al., 2006), an adjusted value of mid-sleep on work-free (weekend) days (MSF) that accounts for possible sleep debt was used to estimate one’s chronotype (Roenneberg et al., 2004). The adjusted value (MSF_sc_) is derived according to the following equation:

(3) $${\text{MSF}}_{\text{sc}}=\text{MSF}-0.5\,\left[{\mathrm{TS}}_{\text{F}}-\left(5\,\left({\mathrm{TS}}_{\text{w}}\right)+2\,\left({\mathrm{TS}}_{\text{F}}\right)/7\right)\right],$$

where TS_w_ is the average total sleep duration (in minutes) on work days and TS_F_ is the average total sleep duration (in minutes) on free days. This equation yields a time (ex. 04:00 h) corresponding to the midpoint of the individual’s sleep cycle. Midpoints earlier than 05:00 h typically denote an earlier chronotype and later midpoints denote a later chronotype (Roenneberg et al., 2003b).

Alertness scores were derived for each testing session. Mean reaction times on the PVT were calculated per participant for correct response trials. A score from 1 to 10 on the VAS at each testing session per participant was analyzed, with higher scores indicating greater alertness.

## Results

### Time of Day Effects in Music Performance

**FIGURE 1:**
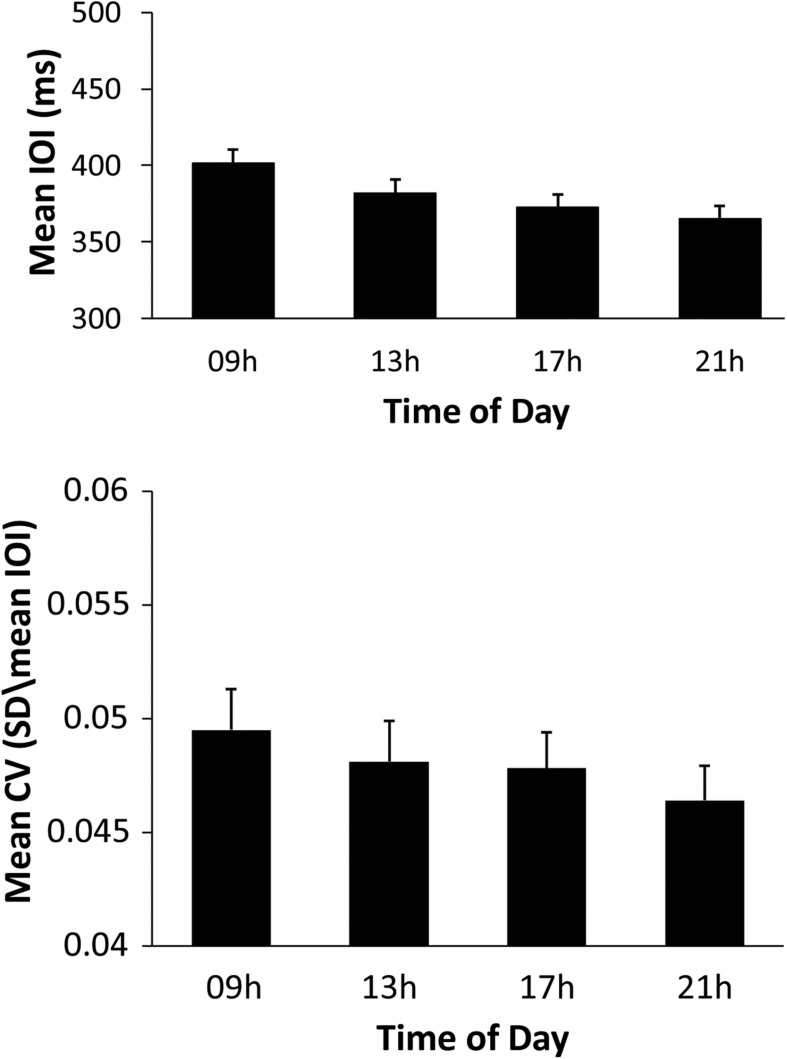
Performers’ mean Spontaneous production rates (ms) by Time of Day **(top)** and mean CV by Time of Day **(bottom)** for all melody performances.

To test for differences in mean SPR values across the day, a two-way ANOVA on mean SPR by Time of Day (09, 13, 17, 21 h) and Melody (Familiar, Unfamiliar) was performed. This analysis indicated significant main effects of Time of Day [*F*(3,93) = 17.42, *p* < 0.01, $\eta_{\text{p}}^{2}$ = 0.36), and of Melody [*F*(1,31) = 41.73, *p* < 0.01, $\eta_{\text{p}}^{2}$ = 0.57], and no significant interaction. Shown in Figure 1 (top), mean SPR was significantly slower at 09 h than at all other testing sessions, and was slower at 13 h than at 21 h (Tukey’s *HSD* = 13.72, *p* < 0.05). SPR was faster for the Unfamiliar melody performances (mean = 362.61 ms) than the Familiar performances (mean = 397.84 ms); this finding is not surprising as the Familiar melody’s rhythm contained half and quarter notes which constrained the fastest rate possible, whereas the Unfamiliar melody contained only quarter notes.

To test whether the stability of music performance changed over the day, the same ANOVA was conducted on mean CV. There were significant main effects of Time of Day [*F*(3,93) = 3.827, *p* = 0.012, $\eta_{\text{p}}^{2}$ = 0.11] and of Melody [*F*(1,31) = 9.200, *p* = 0.005, $\eta_{\text{p}}^{2}$ = 0.23], and no significant interaction. Figure 1 (bottom) shows the CV values; the CV at 09 h was significantly larger than at 17 and 21 h, and the CV at 13 h was significantly larger than at 21 h (Tukey *HSD* = 0.002, *p* < 0.05). Paralleling the findings of mean SPR becoming faster across the day, pianists became more stable in their performances across the day. The mean CV for Familiar melody performances (mean = 0.05) was greater than for Unfamiliar melody performances (mean = 0.045), consistent with the varying rhythmic structure of the Familiar melody compared with the isochronous rhythm of the Unfamiliar melody. Overall, these findings suggest a 09 h effect on SPR and CV that diminished over the day.

To examine whether performers’ alertness levels varied over the testing sessions, we tested participants’ reaction times on correct trials in the Psychomotor Vigilance Task in a one-way ANOVA by Time of Day (09, 13, 17, and 21 h). Mean reaction times varied significantly across the day [*F*(3,93) = 3.70, *p* < 0.01, $\eta_{\text{p}}^{2}$ = 0.11]. Mean reaction times at 09 h were significantly slower (mean = 233.57 ms) than mean reaction times at 21 h (mean = 224.50 ms) (*HSD* = 7.29, *p* < 0.05). No other time-of-day comparisons were significant. In line with the primarily late chronotype sample, these findings suggest that participants were less alert at 09 h than at 21 h. Mean subjective alertness scores (Visual Analog Scale, VAS) did not show significant effects of time of day.

The sublingual body temperatures were assessed with a one-way ANOVA by Time of Day (09, 13, 17, and 21 h). There was a significant main effect [*F*(3,93) = 6.28, *p* = 0.001, $\eta_{\text{p}}^{2}$ = 0.17]; *post hoc* analyses indicated that body temperature at 09h was significantly higher (mean = 36.73°Celsius) than body temperature at 13 and 17 h (*HSD* = 0.237, *p* < 0.05), with no other comparisons differing significantly. Consistent with previous work (Christie and McBrearty, 1979; Monk, 2005) participants’ sublingual temperature decreased slightly in the middle portion of the day and rose again through the evening.

### Individual Differences in Performance Tempo

**FIGURE 2:**
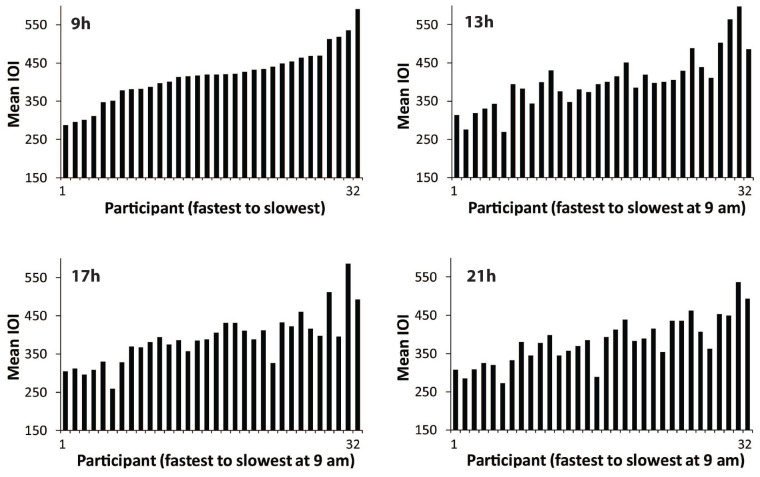
Distributions of performers’ mean SPR values (mean IOI in ms) for the Familiar melody, ordered at each testing session from fastest to slowest performer according to 09 h. Each bar = one performer.

**FIGURE 3:**
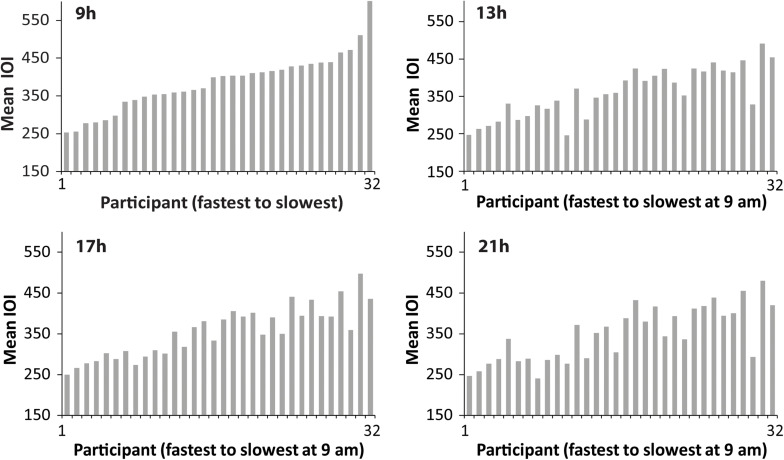
Distributions of performers’ mean SPR values (mean IOI in ms) for the Unfamiliar melody, ordered at each testing session from fastest to slowest performer according to 09 h. Each bar = one performer.

Next, we examined individual differences in spontaneous production rate (SPR). Figure 2 shows the mean spontaneous rates of individuals’ Familiar melody performances at each testing session, ordered in each graph from fastest to slowest individual at 09 h. The similarity of the faster-to-slower patterns across the four graphs suggests that the individual differences in performance tempo were consistent. To test whether the SPR values were stable across times of day, Spearman’s rank order correlations were applied to test whether the ordering of individuals at the 09 h session matched the ordering at the 13, 17, and 21 h sessions. The rank-ordered SPR values held from the 09h session to each testing session [13 h ρ = 0.87, *p* < 0.01; 17 h ρ = 0.85, *p* < 0.01; 21 h ρ = 0.82, *p* < 0.01). Figure 3 shows the same pattern of individuals’ SPR values across testing sessions for the Unfamiliar melody performances, where each graph is again ordered by fastest to slowest individual at 09 h. Similar to the Familiar melodies, the individual differences at 09 h were significantly retained across all testing sessions (13 h ρ = 0.84, *p* < 0.01; 17 h ρ = 0.88, *p* < 0.01; 21 h ρ = 0.81, *p* < 0.01). These findings suggest that large individual differences in spontaneous rates existed for both familiar and unfamiliar melodies, and the individual differences were consistent across times of day.

To address whether individual differences in SPR were related to chronotype, we computed participants’ chronotype from the Munich Chronotype Questionnaire (MCTQ), following Equation 3. The mean and median midsleep point on free days (MSF_sc_) were 05 h14 and 05 h08, respectively (range = 03 h13 to 09 h21). Individuals with MSF_sc_ values later than about 05 h are typically considered a late chronotype or night owl (Roenneberg et al., 2003b). The present sample MSF_sc_ was positively skewed, with 3 of 32 participants in the <04 h range, 23 of 32 participants in the 04–05 h range, and 6 of 32 participants in the ≥06 h range. Although chronotype appears nearly normally distributed in the general population, the overrepresentation of night owls in the present sample is consistent with previous findings for this age group (Roenneberg et al., 2003b) as well as for musicians (Gjermunds et al., 2019). Due to the lack of variability in chronotype and the overrepresentation of night owls, the relationship between chronotype and SPR could not be assessed; the three earliest chronotypes and the six latest chronotypes did not show SPR patterns that differed from the remaining cohort.

We next examined the individual differences in spontaneous rates (mean SPR) in terms of amount of musical training and alertness (reaction times on correct trials from the PVT) using a multiple regression model that predicted mean SPR from years of musical training and reaction time (RT). The multiple regression fits for the Familiar melody performances, predicting mean SPR from RT and Musical Training, were significant at 09 h (*R* = 0.523, *p* < 0.01) and at 13 h (*R* = 0.51, *p* < 0.05). Semi-partial correlations indicated significant contributions to the SPR of both RT (standardized coefficient = 0.35, *p* = 0.035) and Musical Training (standardized coefficient = −0.378, *p* = 0.02) at 09 h. The semi-partial correlations at 13 h indicated similar contributions of RT (standardized coefficient = 0.35, *p* = 0.04) and Musical Training (standardized coefficient = −0.3305, *p* = 0.04). At both 09 and 13 h, individuals’ slower tempi were associated with longer RT values in the PVT (lower alertness) and with less musical training. The same multiple regression model did not predict individuals’ SPR values at 17 h or at 21 h. The same multiple regression model fit to mean SPR values for the Unfamiliar melody performances showed similar influences of alertness (RT) but not of musical training. The multiple regression fit reached significance at 09 h (*R* = 0.45, *p* = 0.04) but not at any other testing session. The semi-partial correlations indicated significant contributions of RT at 09 h (standardized coefficient = 0.3958, *p* = 0.024). Consistent with performances of the Familiar piece, participants with lower alertness scores (higher RT values) performed the Unfamiliar melody at a slower tempo at the first session of the day.

There was no significant relationship between acute sleep deprivation (average duration of sleep in 1 week – duration of single night sleep preceding laboratory session) and individual SPR values at any testing session, for Familiar or Unfamiliar melody performances, suggesting that individual differences in SPRs were not accounted for by differences in acute sleep deprivation.

### Cardiac Dynamics During Music Performance

**FIGURE 4:**
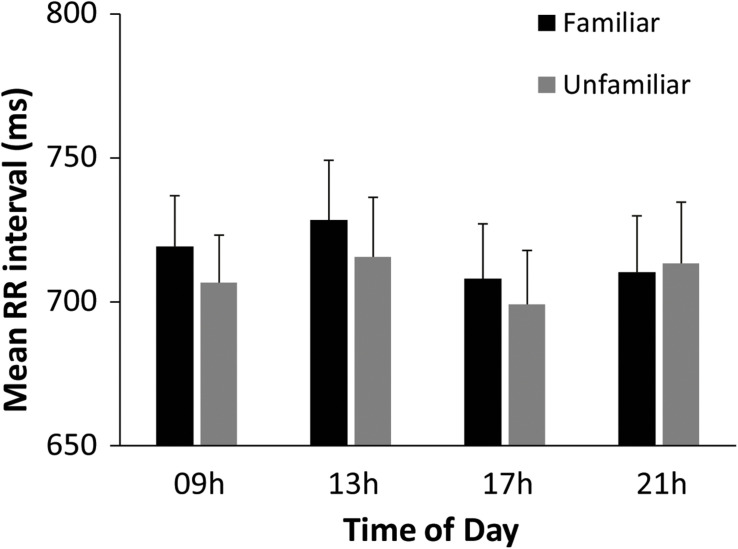
Performer’s mean heartbeat interval (RR interval, in ms) by Time of Day and Melody.

Linear cardiac measures (RR interval and SDNN) were examined to identify whether cardiac activity varied across the day and across music and rest. A two-way within-subjects ANOVA on mean RR interval by Time of Day (09, 13, 17, and 21 h) and Task (Baseline rest, Music Performance) showed a significant main effect of Task [*F*(1,31) = 13.51, *p* = 0.001, $\eta_{\text{p}}^{2}$ = 0.30), and no main effect of Time of Day or interactions. RR interval was shorter during music performance (mean = 712.57 ms) than during baseline rest (mean = 734.12 ms), indicating that pianists’ heart rate increased from baseline to music performance. To examine the two melodies performed at each testing session, a follow-up two-way ANOVA on mean RR interval by Time of Day (09, 13, 17, 21 h) and Melody (Familiar, Unfamiliar) was performed. There was a significant main effect of Melody [*F*(1,31) = 6.27, *p* = 0.02, $\eta_{\text{p}}^{2}$ = 0.17] and a significant Time of Day × Melody interaction [*F*(3,93) = 3.20, *p* = 0.03, $\eta_{\text{p}}^{2}$ = 0.09]. As seen in Figure 4, participants’ RR intervals were shorter during Unfamiliar melody performances than during Familiar melody performances at 09, 13, and 17 h, but not at 21 h (*HSD* = 4.36, *p* < 0.05). Participants’ heart rate increased during the Unfamiliar melody performance relative to the Familiar melody performance earlier in the day but not later in the evening. Similar analyses on mean SDNN values showed no significant effects of time of day or type of melody, and no interaction.

**FIGURE 5:**
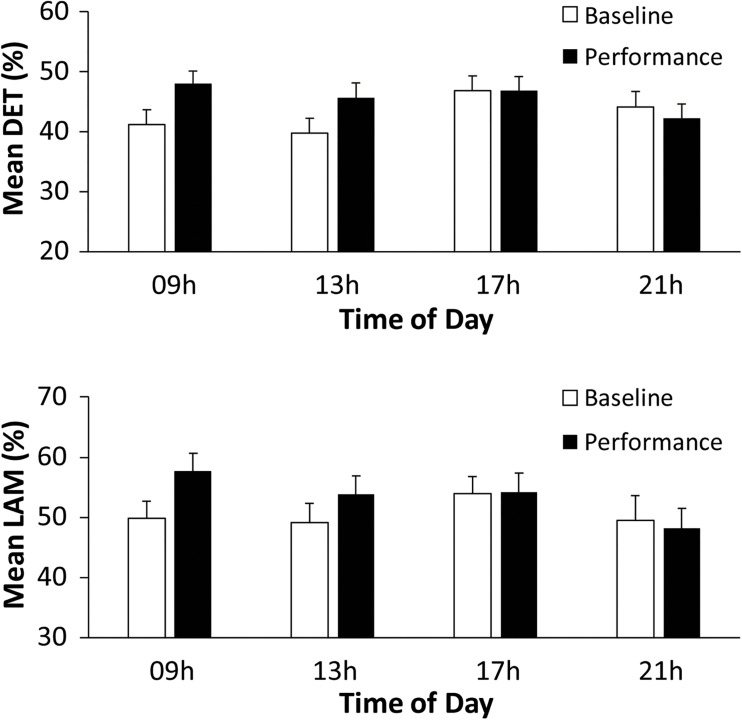
Performers’ mean% Determinism **(top)** and mean% Laminarity **(bottom)**, by Time of Day and Task (Baseline rest/Music performance).

**FIGURE 6:**
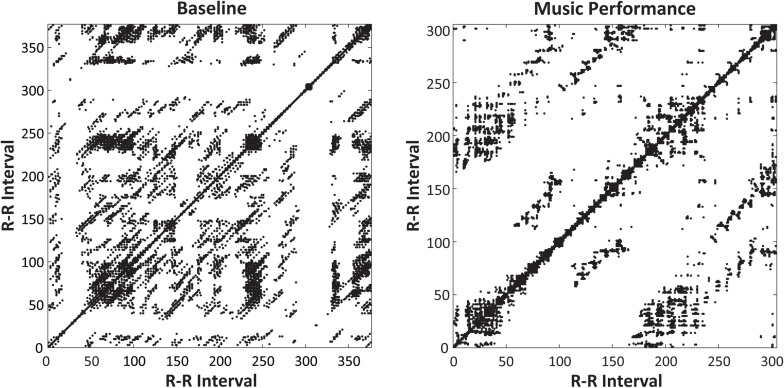
Recurrence plots for a single participant’s heartbeat series (RR intervals, in ms, plotted on *x*- and *y*-axes) at 09 h. Plots include 5-mine Baseline rest period **(left)** and Unfamiliar melody performance **(right)**. The melody performance showed greater determinism (%DET = 58.88) than did the Baseline Rest (%DET = 39.62).

Non-linear RQA measures of cardiac activity evaluated the predictability of performers’ heart rate measures (R–R intervals, in ms). A two-way ANOVA on mean determinism (%DET, measuring predictability) by Time of Day (09, 13, 17, and 21 h) and Task (Baseline rest, Music Performance) showed no main effect of Time of Day, a significant main effect of Task [*F*(1,31) = 4.15, *p* = 0.05, $\eta_{\text{p}}^{2}$ = 0.12] and a significant Time of Day × Task interaction [*F*(3,93) = 6.48, *p* < 0.001, $\eta_{\text{p}}^{2}$ = 0.17]. There was greater determinism (predictability) during music performance (mean %DET = 45.699) than during baseline rest (mean %DET = 42.959). Figure 5 (top) demonstrates that the cardiac activity showed significantly greater determinism during music performance at 09 and 13 h (*HSD* = 0.053, *p* < 0.05) but not at 17 and 21 h. Recurrence plots for a single participant at 09 h in Figure 6 demonstrate the greater amount of determinism or predictability during music performance than during baseline rest.

**FIGURE 7:**
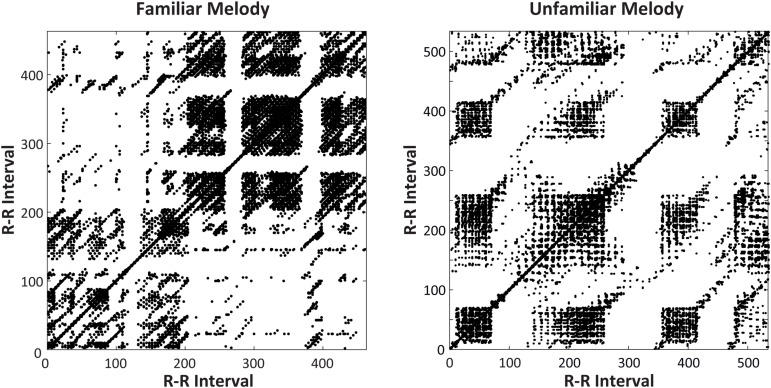
Recurrence plots for a single participant’s heartbeat series (RR intervals, in ms, plotted on *x*- and *y*-axes) at 13 h. Plots include Familiar melody performance **(left)** and Unfamiliar melody performance **(right)**. The Unfamiliar melody performance showed greater Determinism (=53.31%) than did the Familiar melody performance (= 44.04%).

A follow-up two-way ANOVA on mean %DET by Time of Day (09, 13, 17, and 21 h) and Melody (Familiar, Unfamiliar) showed a significant main effect of Melody [*F*(1,31) = 6.348, *p* = 0.017, $\eta_{\text{p}}^{2}$ = 0.17], and no main effects or interactions with Time of Day. Specifically, %DET values were larger during the Unfamiliar melody performances (mean = 46.99) than the Familiar melody performances (mean = 44.41). Figure 7 shows a pair of recurrence plots illustrating this difference for a single subject, where a greater proportion of recurrent points form diagonal lines in the plot on the right (Unfamiliar performance). Thus, greater determinism (predictability) in cardiac activity was seen during music performances compared to rest, and for Unfamiliar melody performances compared to Familiar melody performances.

**FIGURE 8:**
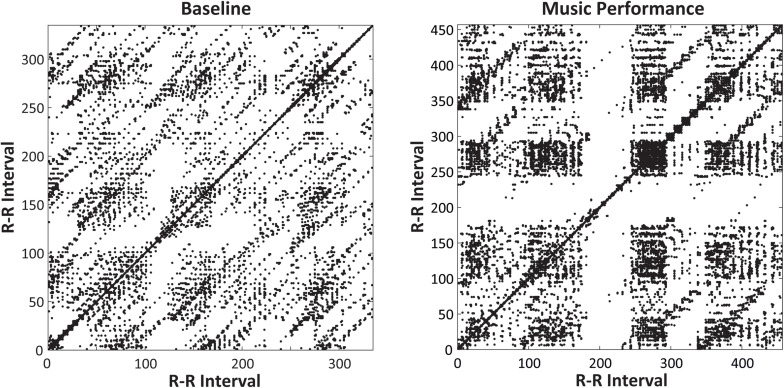
Recurrence plots for a single participant’s heartbeat series (RR intervals, in ms, plotted on *x*- and *y*-axes) at 09 h. Plots include Baseline rest **(left)** and Unfamiliar melody performance **(right)**. The Melody performance showed greater Laminarity (=51.6%) than did the Baseline Rest (= 27.0%).

The same analyses were performed to identify whether laminarity in the cardiac system (how much the system got stuck in a recurrent state) changed with Time of day and Task (Baseline rest and Music Performance). The mean laminarity (LAM) values indicated a significant main effect of Task [*F*(1,31) = 5.415, *p* = 0.027, $\eta_{\text{p}}^{2}$ = 0.15], no main effect of Time of Day, and a significant Time of Day × Task interaction [*F*(3,93) = 3.678, *p* = 0.015, $\eta_{\text{p}}^{2}$ = 0.11]. Recurrence plots for a single participant in Figure 8 show that a greater proportion of points form vertical/horizontal lines during the melody performances (mean %LAM = 53.74) than during baseline rest (mean %LAM = 50.25). *Post hoc* comparisons of the interaction showed that mean laminarity values were significantly greater during music performance than baseline only at 09 h (*HSD* = 0.055, *p* < 0.05), also shown in Figure 5 (bottom). A follow-up ANOVA on mean LAM value by Time of Day and Melody (Familiar, Unfamiliar) showed a significant main effect of Time of Day [*F*(3,93) = 4.107, *p* = 0.009, $\eta_{\text{p}}^{2}$ = 0.17] and no effects or interactions with Melody. Overall, there was greater laminarity and determinism (predictability) in cardiac rhythms during music performance than during baseline rest; that difference was larger at earlier testing sessions. In addition, there was greater determinism during Unfamiliar melody performances than during Familiar melody performances, controlling for time of day.

## Discussion

This study examined time-of-day effects on musicians’ performance tempo for simple melodies, and whether circadian effects on physiology could account for individual differences in performance tempo. Trained pianists’ performance rates for familiar and unfamiliar melodies were recorded at four testing sessions in a single day (09, 13, 17, and 21 h) while cardiac activity was recorded. Resting measures of performers’ cardiac activity, alertness, and body temperature were recorded at each testing session. Additionally, this study utilized a non-linear analysis technique (RQA) to investigate cardiac dynamics during music performance both within and across times of day.

Overall, musicians’ spontaneous performance rates were slower and more variable at 09 h and became slightly faster and less variable at later testing sessions. The largest difference in SPR and variability of performances was between 09 and 21 h, similar to previous findings on spontaneous motor rates of tapping (Dosseville et al., 2002) and cycling (Moussay et al., 2002), which have shown slowest rates in the morning and fastest rates in the evening. These results suggest that melody performances increased in tempo and in temporal regularity from the morning to the evening, a finding that is somewhat consistent with a sample of largely night-owl chronotypes (Van Vugt et al., 2013). Participants completed all testing sessions in the same order in this study (to control for sleep differences between testing sessions); therefore, it is possible that some changes in melody performance rate and temporal variability were attributable to practice effects over the session trials. In the context of motor sequencing, performing repeated trials of specific finger sequences in a blocked (rather than randomized) fashion typically results in faster learning rates (Fogel et al., 2017; Caramiaux et al., 2018). The observed changes in participants’ melody performances across times of day were similar for unfamiliar and familiar melodies, which is consistent with practice effects over trials (as opposed to familiarity with the musical melodies).

Musicians showed large individual differences in spontaneous performance rates (SPR), replicating previous studies on natural movement rates in music performance (Zamm et al., 2015; Palmer et al., 2019) and tapping tasks (Scheurich et al., 2018). Importantly, the individual differences in pianists’ performance tempo were consistent across the day for both familiar and unfamiliar melodies: Pianists who performed quickly in the morning also performed quickly in the evening, and the same was true for pianists with slower rates. These findings are consistent with dynamical systems theory predictions that an individual’s natural movement rate, a property of a periodic oscillatory system (Kelso, 1997), may serve as an attractor state at which movement efficiency is maximized (Hoyt and Taylor, 1981; Zamm et al., 2018). Indeed, neuromuscular fatigue has been shown to be minimized at cyclists’ spontaneous (uncued) pedaling rates for a given load resistance (Takaishi et al., 1996; Moussay et al., 2002), and reduced kinetic energy expenditure in pianists’ finger movements is associated with increased temporal accuracy of performance (Goebl and Palmer, 2014). Our finding of consistency across testing sessions in individuals’ performance tempo suggests that one’s spontaneous production rate may be an energy-efficient state for melody performance that transcends time of day effects or familiarity with the melody.

Alertness measures also showed time of day effects and explained some of the individual variability in performance rates; participants who performed melodies at a slower rate at 09 and 13 h had slower reaction times on the PVT task at these times. Lower alertness in the morning is not surprising for the later chronotype sample of musicians tested here (Roenneberg et al., 2003a). At early testing times, musicians’ spontaneous performance rates were influenced by both physiological (alertness) and behavioral (musical training) variables. Participants with faster reaction times in the PVT task and more years of formal piano training tended to show faster performance rates at 09 and 13 h. Interestingly, neither physiological nor behavioral variables predicted performance rates later in the day. Alertness and musical training may have greater effects on melody performance when musicians are less comfortable with a musical task (for example at the first 09 h testing session), an interpretation consistent with the general increased temporal stability reported for musicians with increased training (Scheurich et al., 2018). This hypothesis could be addressed by randomizing participants’ first testing session to begin at different times of day in future studies.

Finally, the complexity of musicians’ cardiac activity was compared between 5-min rest periods and music performances, as well as between performances of familiar and unfamiliar melodies. Both linear and non-linear measures of heart rate (R–R intervals) indicated significant differences from rest to music performance, with faster and more patterned (deterministic) heart rates during music performance than during rest, across times of day. The largest differences between music performance and rest were seen at 09 h and at 13 h. In addition, heart rates were faster during performances of unfamiliar melodies than familiar melodies, and laminarity (recurring patterns) of cardiac activity was greater for unfamiliar melodies than for familiar melodies. Increased predictability of cardiac signals has been observed during increases in task difficulty for both physical (Javorka et al., 2009; Konvalinka et al., 2011; Schlenker et al., 2016) as well as cognitive behaviors (Goshvarpour and Goshvarpour, 2012). Overall, the differences in cardiac dynamics between rest and music performance, and between performance of familiar and unfamiliar melodies, suggest that increased predictability and stability of cardiac signals may be a physiological marker of increased behavioral difficulty.

The current findings were limited by the simple musical materials used, and the chronotype sample of musicians obtained. Two simple melodies were included to reduce the memorization demands on participants; those melodies had simple but not identical rhythmic structures. Future research may examine the roles of musical performance styles and rhythmic complexity in performance rates and cardiac rhythms. Furthermore, the chronotype of the obtained musician sample was biased toward night owls, in line with previous research (Gjermunds et al., 2019). It is possible that decreases in SPR and increases in performance stability over the day were specific to the night owl chronotype, as late chronotypes perform better on strength tasks (Tamm et al., 2009) and music performance tasks (Van Vugt et al., 2013) in the evening relative to the morning. Future research may extend these findings to a more diverse sampling of chronotypes.

In sum, pianists’ rates of melody performances increased and variability decreased across the 12 h day, similar to circadian influences on other motor skills. Time of day may be an important relationship for musicians to consider; there may be ideal times of day to practice or perform. Individual differences in performance rates early in the day were predicted by both alertness and musical training. In addition, large individual differences in the musicians’ performance rates remained consistent across the 12-h time period. Finally, pianists’ cardiac dynamics became more predictable and recurred more during music performance than during a baseline rest interval, as well as during performances of an unfamiliar melody than a familiar melody. To our knowledge, these findings provide the first evidence that performing music affects non-linearities of cardiac dynamics in specific and replicable ways within individuals. Overall, these discoveries of performers’ cardiac dynamics suggest possible applications to music therapy; the time of day at which music is performed, as well as the familiarity of the music, may influence music’s ability to modulate physiological systems.

## Data Availability Statement

The raw data supporting the conclusions of this article will be made available by the authors, upon reasonable request.

## Ethics Statement

The studies involving human participants were reviewed by the McGill University Research Ethics Board. The patients/participants provided their written informed consent to participate in this study.

## Author Contributions

SW and CP designed the experiments, wrote and edited the manuscript. SW conducted the experiments and analyzed the data. All the authors contributed to the article and approved the submitted version.

## Conflict of Interest

The authors declare that the research was conducted in the absence of any commercial or financial relationships that could be construed as a potential conflict of interest.
